# Effects of Health Anxiety, Social Support, and Coping on Dissociation with Mediating Role of Perceived Stress during the COVID-19 Pandemic

**DOI:** 10.3390/ijerph20085491

**Published:** 2023-04-12

**Authors:** László Róbert Kolozsvári, Viktor Rekenyi, Szabolcs Garbóczy, Ágnes Hőgye-Nagy, Anita Szemán-Nagy, Mohamed Sayed-Ahmad, Katalin Héjja-Nagy

**Affiliations:** 1 Doctoral School of Health Sciences, University of Debrecen, 4032 Debrecen, Hungary; 2 Department of Family and Occupational Medicine, Faculty of Medicine, University of Debrecen, 4032 Debrecen, Hungary; 3 Department of Psychiatry, Faculty of Medicine, University of Debrecen, 4032 Debrecen, Hungary; 4 Department of Work and Social Psychology, Institute of Psychology, University of Debrecen, 4032 Debrecen, Hungary; 5 Department of Personality and Clinical Psychology, Institute of Psychology, University of Debrecen, 4032 Debrecen, Hungary; 6 Department of Radiology and Imaging Science, Faculty of Medicine, University of Debrecen, 4032 Debrecen, Hungary

**Keywords:** perceived stress, social support, health anxiety, coping, lockdown, COVID-19, international students, domestic students

## Abstract

Background: Our study aimed to examine whether health anxiety, social support, and ways of coping relate to dissociation directly or only through the mediation of perceived stress, moderated by the time of measurement (lockdown). We investigated the effect of perceived stress on different forms (sub-scales) of dissociation. Methods: A cross-sectional survey was conducted by an online form at two points in time: the beginning and the later stage of the COVID-19 pandemic. Results: We received a total of 1711 responses. Perceived stress moderately correlated with dissociation in both international and Hungarian samples. Health anxiety showed a strong direct and indirect correlation with dissociation. Regarding social support, the support of family significantly decreased the dissociative experiences in the Hungarian sample mediated by perceived and direct stress. In the international sample, goal-oriented coping strategies strongly decreased all dissociation scales in the first measurement, through the mediation of perceived stress. As for the Hungarian sample, positive thinking was found to decrease dissociation by decreasing perceived stress. Conclusion: health anxiety, coping, and social support appeared to influence dissociation directly and through the mediation of perceived stress. Social support, mainly support of the family and problem-focused coping strategies may decrease the level of stress, this way decreasing dissociative behavior.

## 1. Introduction

On 11 March 2020, the WHO classified COVID-19 a pandemic [1]. To avoid rapid spread, governments and international organizations introduced various measures, including nationwide lockdowns in several countries, and Hungary was no exception [2].

The lockdown was ordered on 28 March 2020 and eventually—after a few extensions—it was lifted in the countryside on 4 May 2020 and in Budapest on 18 May 2020. Among the first cases, there was a large number of Hungarians and foreigners who came home from abroad, e.g., Iranian students. The Hungarian foreign minister said “a new wave of migrants heading towards Europe, presenting health and security risks, is possible due to the pandemic” [2]. Turbid statements such as this one could have caused the birth of, often subconscious, xenophobic tendencies, even in individuals who previously had no such inclinations.

After the initial phase and as vaccines were introduced and spread across the globe, the number of patients treated in hospitals and on ventilators also decreased [2]. At first, the vaccination program was made available only for essential workers and high-risk individuals, but after that, anyone who wanted to receive the vaccine had the opportunity due to Hungary’s sufficient supply of vaccines by then. The head of the prime minister’s office ranked Hungary as one of the safest countries and said “The Hungarian government is ready to mitigate the damage students suffered during the coronavirus epidemic due to the lack of classroom education” [2].

Since then, mental effects of the pandemic have become widely known, as a plethora of studies has been conducted and written about it. These effects include, to name just a few, increased psychological distress, anxiety, depression, and disturbed sleep [3,4,5,6].

Confinement and isolation were already known to have mental and health implications before COVID-19, such as worse cardiovascular and mental health outcomes [7]. Lockdowns and quarantines during the pandemic created more opportunities to examine mental health of people living under these conditions, and thus the number of studies also increased expeditiously. In the course of the pandemic, confinement and isolation were found to influence important aspects of everyday life, such as food supply, teaching, and travel; they have a significant effect on the appearance of depression and anxiety, and their effect on mental health can be extensive and long-lasting [8,9,10].

That is why we found it important to examine psychological and social constructs, such as social support, perceived stress, health anxiety, coping mechanisms, and dissociation, during COVID-19 and even more so during the lockdowns. The relationship between perceived stress and dissociation is well-documented in the scientific literature. The COVID-19 pandemic increased perceived stress. We aimed to study variables that are closely related to perceived stress, such as coping, health anxiety, and social support. Al-though studies have been conducted for these separately, and even some have been combined, there has not yet been a study that examined all of them together to the best of our knowledge. We are now trying to fill this gap with this study. Our main focus (dependent variable) was dissociation, because in traumatic situations dissociation is a significant phenomenon, and it may lead to severe consequences such as PTSD [11] and addictive behavior [12]. According to recent studies, the importance of dissociation in mental health is under-recognized [13].

We aimed to examine the following: how perceived stress is related to dissociation, and how health anxiety, social support, and ways of coping relate to dissociation directly and through the mediation of perceived stress, moderated by the time of measurement (lockdown). Researchers found that higher levels of the psychological constructs we examined (perceived stress, anxiety, exhaustion, and depression) can be detected at a higher degree in those participating in higher education [3,14,15,16,17].

Regarding dissociation, we followed in the footsteps of Janet, who was the first to study dissociation comprehensively and in detail and “claimed it as the crucial psychological process with which the organism reacts to overwhelming trauma and which results in the wide variety of symptoms” [18]. He believed that under normal circumstances, the awareness of feelings, emotions, thoughts, and actions connected to a certain event or experience is concentrated in one consciousness and can be controlled voluntarily. However, the memories of frightening or novel events and experiences that do not fit into existing cognitive schemas can detach from consciousness and go beyond the authority of voluntary control, and the details of the unintegrated experiences created in this way can emerge as pathological automatisms [18].

Almost a hundred years later, Janet’s thoughts were carried forward by Bernstein and Putnam, who created the questionnaire we also utilized, the Dissociative Experiences Scale (DES). According to their definition, “dissociation is a lack of the normal integration of thoughts, feelings, and experiences into the stream of consciousness and memory” [19].

Even later, Grabe et al., summarized this as “dissociation is considered to serve as a defense mechanism against intolerable, trauma-associated memories and feelings, and results from a disintegration of consciousness, memory, identity, and perception” [20].

Initially, Janet thought of all forms of dissociation as pathological. With all respect to him, over time his theories have been further scrutinized and it has been found that dissociation is a spectrum with adaptive and pathological forms [21,22,23].

Different authors have described that greater stress and traumatic experiences promote dissociation [24,25]. Moreover, in a large study including 6941 individuals from the general population, Černis et al. [13] explored, using network approaches, how dissociative experiences taking the form of a Felt Sense of Anomaly (FSA) relate to common mental health conditions and psychotic experiences and found that dissociation is connected to many mental health disorders and may influence a number of presentations, particularly psychotic experiences.

Certain studies have already addressed dissociation emerging during COVID-19. In a study conducted on the general population, authors found that women have a higher rate of traumatic distress and that the Internet-addicted subgroup experienced dissociative experiences more often [26]. In another study, authors found that hopelessness is associated with more dissociative experiences, which contribute to the strengthening of denial about COVID-19, which is a maladaptive defense against feelings of hopelessness [27]. In the study conducted in Italy with an online questionnaire during the lockdown, it was possible to prove the direct relationship between the pathological personality (subjects with previous psychopathological vulnerability) and the fear of COVID-19, and that although dissociation and emotion dysregulation were not involved in the relationship between the two as intervening mediators. However, they found that dissociation and emotion dysregulation play an important role in the relationship between pathological personality and PTSD [28]. A French study of medical staff found that, among others, levels of peritraumatic dissociation were lower for men compared to women [29].

In these studies, different aspects of dissociation (the questionnaire subscales, namely absorption, depersonalization, and amnesia) were not differentiated, rather a summated score was used—however, different dissociative experiences may be different in their adaptiveness in stressful situations.

Among many psychological consequences of the pandemic, our main focus was dissociation caused by perceived stress. Why did we focus on dissociation? First, COVID-19 significantly increased addictive behavior in society [12], and as addiction may be considered a dissociative behavior, dissociation has to do with addictions [26]. Second, dissociative experiences entail a higher risk of the appearance of dissociative disorders such as PTSD [11]. Stress promotes dissociation, and the relationship between stress and dissociation is well-documented in the related scientific literature [24,25], but perceived stress in the same pandemic situation may differ along various variables. We aimed to study this relationship during the COVID-19 pandemic and expanded the examination by protective factors (e.g., social support, adaptive coping), and risk factors (e.g., health anxiety, maladaptive coping) as these constructs may have a role in the level of perceived stress.

### 1.1. Perceived Stress

We used this term as Cohen, Kamarck, and Mermelstein defined it: a crucial psychological factor that refers to the degree to which events in a person’s life are perceived as stressful, unpredictable, and uncontrollable [11]. During the COVID-19 pandemic, a wide range of studies was conducted in the area of stress. A meta-analysis of studies examining the effects of the COVID-19 pandemic on mental health found that anxiety has increased in the average population compared to pre-pandemic times [5]. A review emphasizes increased stress and lists which factors may contribute to its development, highlighting uncertainty about the disease and social isolation [4]. Others have found significantly increased levels of distress in the general population [3], especially during the early stages of the pandemic [14]. Examining the impact of the lockdown, it was found among students that the value of perceived stress increased, while the quality of mental well-being and the extent of physical exercise decreased during lockdown [30].

High levels of perceived stress appear to elevate dissociative experiences. In a longitudinal study by De Wachter et al., stress levels of participants were reduced by information and psychological support. Their results support a one-directional causal relationship: a decrease in perceived stress leads to a decrease in dissociative phenomena [31].

### 1.2. Social Support

In their 1985 work, Cohen and Syme defined social support as the resources provided by other persons, and they stated that it can have both negative and positive effects on health and well-being. In their work, two models are presented: in one, a direct effect is outlined, in which social support enhances health and well-being regardless of the level of stress; the other model is the buffering hypothesis, in which “social support exerts its beneficial effects in the presence of stress by protecting people from the pathogenic effects of such stress by attenuating or preventing a stress response” [32]. Other authors believe that “neither type of support effect is found uniformly”. Support refers to the positive, potentially health-promoting, or stress-buffering aspects of relationships such as instrumental aid, emotional caring or concern, and information. In essence, supportive relationships directly provide something that people need to stay healthy or to adapt to stress [33].

Authors found that social support can not only play a role in avoiding negative symptoms but can also help with adequate adaptation after COVID-19 and can be an excellent tool in the fight against stress and trauma [34]. In recent literature, we can find evidence that perceived social support moderates the relationship between COVID-19-related anxiety and psychological health [35] and that it can provide a buffering effect on mental health against the negative impact caused by low resilience [36]. Lower levels of perceived social support were found to be significantly associated with elevated risk for depression and poorer sleep quality [37]. Resilience, coping behaviors, and social support were identified as protective factors against loneliness [38].

Several studies examined the interaction between social support and dissociation as well, though results are somewhat contradicting; e.g., Baranyi et al. [39] did not support a link between PTSD and unfavorable perception of supportive relationships, while Coronas et al. [40] found that perceived social deprivation was related to the diagnosis of PTSD 2 months after a motor vehicle accident among victims. In a study, even Dissociative Identity Disorder was found to be best predicted by the absence of social and familial support in combination with an abuse history [41].

### 1.3. Health Anxiety

According to its definition, health anxiety is the way individuals think and behave about their health and the way they experience their concerns and threats to their health. Health anxiety lies on a spectrum, and while it can have a signaling function to initiate appropriate behaviors, it also can reach overwhelming levels with detrimental consequences [42]. However, both extremes can cause harm. AT one extreme, when health anxiety is excessively low, the person ignores their body’s signals and seeks medical help too late or disregards important measures. At the other extreme, when the level of health anxiety is excessively high, the person can overburden the health care system or worry excessively about their health while they remain locked in their rooms, afraid of the contagion [43].

The previously mentioned review also highlights the role of health anxiety on mental health and states that the level of health anxiety may rise during the epidemic [4]. However, it may be interesting that according to a systematic review analyzing longitudinal studies, the level of anxiety only increased in the initial period compared to the times before COVID-19, and later this seemed to settle down [44]. Interestingly, in contrast to this review in which the scientists found that the increase in measures of anxiety and general mental health observed in March–April 2020 decreased from May–July 2020 (the increase in depression remained higher) [44], the review examining college students found that, compared to the early results, the level of anxiety and depression among the students increased [45].

### 1.4. Coping

The dimensions of coping were determined based on Lazarus’ transactional theory of stress. According to this, the definition of coping is how individuals cope with stress. According to Lazarus and Folkman, stress is a relationship between the environment and the person, which a person perceives as exceeding his abilities and threatening his well-being. Coping is nothing more than a variable cognitive and behavioral effort that the person uses to manage internal and/or external demands that exceed the person’s resources, and it does all this by reducing demands or strengthening personal resources to meet demands. Management can refer to the environment or the person, past or future, and can be instrumental or palliative. Two types of coping are distinguished, between which overlaps can be observed: problem-focused coping (changing the problematic environment-person relationship) and emotion-focused coping (regulating emotional distress) [46]. 

In one study, the majority of college students used problem-focused coping, e.g., they followed strict measures, read about COVID, and avoided public places, and only a few used emotional-focused coping, e.g., venting emotions or adopting a positive attitude [47]. In another Austrian study, active coping and social support were highlighted, which helped mental well-being [48]. Interestingly, in a study in which nurses and nurse students were examined, it was found that practicing nurses use problem-focused coping more than students, and this may be because there is a psychological typhoon eye effect which means that people are less affected at the epicenter of the events [49]. Also, in their study, male students were more prone to use emotion-focused coping. As an explanation, they suggest that women are less able to cope emotionally, as they are more emotional, so they can be swept away by their emotions [50]. Overall, it seems that problem-focused coping was found to be more effective and people used it more often. The main contradiction and interesting thing is that most people experienced the virus as unpredictable and uncontrollable, at least initially. Along with this, many people used problem-focused coping, despite the fact that researchers previously found that problem-focused coping can be used primarily in encounters appraised as changeable [51]. On the other hand, it seems certain that avoiding or denying things is not expedient, as those who try to cope in this way have experienced increased levels of anxiety and stress [52].

### 1.5. Gender

Female gender was identified as a risk factor for higher stress levels in several meta-analyses during the pandemic [3,6,15,53] and overall; it was found that the pandemic affected women more deeply mentally than men [54]. According to a Malaysian study, the female gender and being alone as factors were significantly associated with elevated anxiety values among students. Examining individual stressors, they found that financial issues, online education, and an uncertain future or academic career were at the top [55]. The feelings of the female students regarding the quarantine were more negative compared to their male counterparts, and Italian researchers also found differences similar to other nations when examining the students (increased nervousness, irritability, rumination) and concluded that the student population is more vulnerable [56].

In a narrative review, different sensitivity groups were also identified among women, and they found that the pandemic can amplify the inequalities between the sexes and that social support can be a key protective factor in women’s cases [57].

### 1.6. Lockdown

The lockdown posed a great challenge for the entire population; from the food supply and the loss of financial income, through the reduction of body exercise and travel, to gender relations and domestic violence, everything was affected and had its own mental health implications [8].

The effects of lockdown on mental health are now well known, e.g., it can significantly elevate the amount of perceived stress [10]. This is especially true for students. It can be challenging for international students to get housing, they cannot travel home in these difficult times, which can put a special financial burden on them, and those who manage to get home may be uncertain as to whether they will be able to continue their studies [58]. With the closure of universities, students have adopted new habits, often not by choice. Among them was that classes were attended online. These made personal meetings impossible, and therefore it made them less motivated to study harder, to learn and apply interpersonal and social skills, which also became unattainable in such an environment [59]. The lockdown and the COVID-19 epidemic itself significantly increased the level of student suicides compared to previous years, which draws our attention to serious problems [6,60]. Other studies examining the general population note that school closings, the transition to online education, and isolation from others can all have effects on mental health [8].

A meta-analysis regarding the lockdown found minimal but significant effect sizes for anxiety and depression concerning the lockdown, but at the same time, no significant results were obtained regarding social support, and gender was not found to be a significant moderating factor. According to their conclusion, the lockdown causes heterogeneous differences, which are not outstanding in their magnitude, and the majority of people are psychologically resilient to the lockdown [9].

### 1.7. Aims of the Study

The aim of this study is to investigate how coping, social support, health anxiety (independent or predictor variables) affect dissociation (dependent variable) overall and its subscales. We would like to examine if these variables have direct effects on dissociation as well, or if their effect can only be explained by mediation through the effect of perceived stress (as a mediator variable).

## 2. Materials and Methods

### 2.1. Study Design and Setting

When reporting survey design and sample selection, we consider the CHERRIES statement [61]. The survey was approved by the Hungarian Ethical Review Committee for Research in Psychology (see Patents). In our study, participants filled out an online questionnaire voluntarily. Google Forms was used to create the online survey. Data results of the questionnaire is protected by a password that is only known to the leader of the research. No personal data were collected that would allow the identification of subjects. Informed consent was included in the online questionnaire, on the first page, so as the estimated time of completion.

We conducted a cross-sectional survey at two points in time: the first round (Study 1) was conducted at the time of the nationwide lockdown during one of the most stressful periods of the COVID-19 pandemic in Hungary and when the virus was largely unknown; the second round took place after the lockdown (Study 2). Peace prevailed in the country, easing the strict measures, very low positive test ratio, few new cases, and minimal COVID-related deaths. We found that fewer people participated in the later survey, around the time the virus was tamed by knowledge, vaccines, and measurements. During the COVID-19 period, several longitudinal surveys were conducted, and it was a general problem to lose participants from the first round to the further runs [14].

### 2.2. Study Participants and Sampling

The target population of our study was the students at the second largest university in Hungary, the University of Debrecen from all study programs and study levels (undergraduate, graduate, and postgraduate). We used convenience sampling. The students were approached through social media platforms (Facebook^®^) as well as the official administration system at the university (Neptun system). Both domestic and international students were recruited to participate in our survey and the questionnaire was available in Hungarian and English languages. Regarding international students, English fluency is an entry requirement at the University of Debrecen and students have been doing their studies in English too. All participants should have been at least 18 years or older and enrolled in a study program at the University of Debrecen to be eligible for participation on a self-reported base. The questionnaires were administered on 20 April 2020 in the first round (Study 1). The second measurement started on 20 July 2021 (Study 2). Google Forms checked IP addresses, preventing double completion. Partly incomplete questionnaires were included, but the sample sizes of the particular statistics are different because only complete answers were analyzed.

Our sample is not representative, so non-response bias should be taken into account when drawing conclusions, though we do not presume major differences between respondents and non-respondents. 

### 2.3. Survey Instruments

Our survey questionnaire has solicited anonymous responses using brief sociodemographic items and international scales, namely, the Perceived Stress Scale (PSS) [62,63,64], the Multidimensional Scale of Perceived Social Support (MSPSS) [65,66], the Short Health Anxiety Inventory (SHAI) [67,68], the Ways of Coping Questionnaire (WCQ) [69,70,71] and the Dissociative Experiences Scale (DES) [19,72,73]. The sociodemographic questions were about age, gender, and faculty/study program. Questions were not randomized, they followed the same order for every participant.

#### 2.3.1. The Perceived Stress Scale (PSS)

To assess the stress encountered by the students, we used the Perceived Stress Scale (PSS) which was designed by Cohen et al. [62] and it asks about the stressful situations people went through in the preceding month. It contains 10 statements that respondents can answer on a 5-point (0–4) Likert scale [63]. Among the Hungarian university students, we used the Hungarian version of the PSS [64], which differs from the English version in that it contains 14 statements [64].

#### 2.3.2. Multidimensional Scale of Perceived Social Support (MSPSS)

To measure the amount of social support the students felt they receive we used the Multidimensional Scale of Perceived Social Support (MSPSS). The 12 items are rated on a 7-point Likert scale in the English version [65] while the 10-item Hungarian version uses a 5-point Likert scale [66]. The higher score can be interpreted as a greater amount of available social support. Three subscales were identified, each addressing a different source of support: Family, Friends, and Significant Others [65,66].

#### 2.3.3. Short Health Anxiety Inventory (SHAI)

The third scale we utilized was the Short Health Anxiety Inventory (SHAI), which has 18 items and two subscales. The first subscale comprises 14 items that examine to what degree the respondents were worried about their health, about a serious illness, and about their bodily sensations in the past six months and what their environment said, how much attention they paid to their health. The second subscale of SHAI comprises 4 items that try to evaluate the negative consequences of the illness if it occurs [67]. There are 4 statements for every item in an increasing frequency order (scored from 0 to 3) and one of the four statements must be chosen [74]. For the Hungarian students, the validated Hungarian version of the SHAI was used [68]. The scoring differs from the original version in that the four statements are scored from 1 to 4, but the statements themselves are the same [68].

#### 2.3.4. Ways of Coping Questionnaire (WCQ)

To see what coping strategies the students employ to relieve stress, we used the 26-Item Ways of Coping Questionnaire (WCQ) which was based on the revised 66-Item version [69] and developed by Sørlie and Sexton [70]. The international students answered the validated English version of the 26-Item WCQ that examines how often the respondents used certain coping mechanisms in recently occurring stressful situations. The responses are scored on a 4-point Likert scale (from 0 = “does not apply and/or not used” to 3 = “used a great deal”). The WCQ distinguishes five different factors: Seeking support, Goal-oriented, Thinking it over, Wishful thinking, and Avoidance [70]. 

For the Hungarian students, we utilized the validated Hungarian 16-Item WCQ [71]. The authors identified four dimensions: Cognitive restructuring/Adaptation, Problem analysis, Stress reduction, and Helplessness/Passive coping [71]. 

#### 2.3.5. The Dissociative Experiences Scale (DES) 

This scale was developed by Carlson and Putnam to measure dissociative experiences. During the design of DES, the scale was defined as a continuum of dissociative experiences based on the number and frequency [19]. On this continuum, healthy people report rare and few dissociative experiences (i.e., when is rarely and few are the different dissociative experiences the person experiences, they can even be healthy experiences). According to Vanderlinden, the dissociative experiences that are considered to be healthy originate from the adaptive dissociative mechanisms which are absorption and loss of control [75]. As the scale moves towards the other endpoint, more and more individuals can be found who have clinical symptoms and report diverse and varied dissociative experiences [19]. The number and frequency of symptoms make it likely that the clinical condition will appear. The DES is a series of 28 statements of questions that describe dissociative symptoms in general and could initially be marked on a scale (DES-I) [19] how often a person feels the given symptom in a part of their everyday life, and using the updated version the same could be done on a 0% to 100% scale (DES-II) [74]. The average of the summation gives how many points the person has completed on DES. The higher it is the more likely the presence of dissociative symptoms. Some dissociative activity can also be measured among healthy people, which was found to be 4.38 points [19]. This value was found to be the highest in dissociative identity disorder (57.1), while it was found to be the second-highest in post-traumatic stress disorder, PTSD (31.3) [76]. The test has three subscales: amnestic dissociation; absorption and imagination; and depersonalization and derealization [72,73].

#### 2.3.6. Data Analysis

We calculated the mean scores of the items for the aggregated measures of the Perceived Stress Scale, Short Health Anxiety Inventory, and Dissociative Experiences Scale. For the Multidimensional Scale of Perceived Social Support and the English version of the Ways of Coping Questionnaire, means were calculated for each subscale. In the case of the Hungarian version of WCQ, the authors of adaptation suggested investigating the factor structure of used data [71]. Since Cronbach’s alphas were unacceptable (below 0.5) for 3 subscales, we conducted exploratory factor analysis with the ‘Minimum residual’ extraction method and ‘oblimin’ rotation. Bartlett’s Test of Sphericity was significant with χ^2^ (120) = 332, *p* < 0.001, KMO Measure of Sampling Adequacy was 0.821 for the four-factor solution (Table 1).

Factor 1 consisted of items connected to positive thinking, Factor 2 contained items connected to distancing coping, Item 2 in Factor 3 is connected to humor, while the last factor had items connected to taking up outer perspectives. Mean scores were computed according to the factors, except for humor coping, for which the single item was kept. Descriptive statistics and reliability measures can be seen in Table 2 and Table 3. Cronbach’s alpha measure of reliability had good or excellent values for most scales, acceptable values for wishful thinking and thinking it over coping in the international sample, positive coping in the Hungarian sample, questionable for outer perspective coping, and poor values for avoidant coping in the Hungarian sample.

RStudio and IBM SPSS 27.0 were used to analyze the data. Mann-Whitney U-test was used to investigate the differences between the two measurements and between males and females. Spearman correlation was used for examining connections between variables.

We used conditional process modeling by Hayes (2018) to test the independent effects of health anxiety, social support and coping on dissociation, and the mediating role of perceived stress [77]. We also tested the moderating effect of measurement time, which was dummy coded and we used model 8. We used health anxiety, social support and coping scales as predictor variables, always one predictor at a time, other variables were set as covariates. Bootstrapping was used with 5000 samples and the same seed across models. We used the PROCESS syntax file provided on www.processmacro.org (accessed on 12 September 2022).

## 3. Results

We received a total of 1711 responses, but *n* = 29 respondents were excluded for not staying in Hungary during the lock-down period, and *n* = 2 respondents were excluded, because they answered only the first questions in the questionnaire. In the final sample, there were *n* = 483 (*n* = 347 in the first measurement, *n* = 136 in the second measurement) valid responses from international and *n* = 1197 (*n* = 950 in the first measurement, *n* = 247 in the second measurement) valid responses from Hungarian students. Of the international students, 58.3% were females, and 41.5% were males, with age *M =* 22.67 and *SD =* 4.344. Of the Hungarian students, 75% were females, and 25% are males, with age *M =* 24.98 and *SD* = 8.047. As the questionnaire was somewhat different in English and in Hungarian, we divided our sample and analyzed the two groups separately.

### 3.1. Descriptive and Correlational Analysis

In the international sample, gender differences were found regarding absorption (U_int_abs_ = 24.140, *p* < 0.05); females reported higher values (MD_int_absf_ = 32.5; MD_int_absm_ = 25). Amnesia and depersonalization showed statistically higher values in the second measurement (U_int_amn_ = 19.951, p_int_amn_ < 0.05, MD_int_amn1_ = 5; MD_int_amn2_ = 10; U_int_dep_ = 20.351, p_int_dep_ < 0.05, MD_int_dep1_ = 3.33; MD_int_dep2_ = 10). In the Hungarian sample, males had significantly higher values in amnesia (U_hun___amn_ = 120.173, p_hun___amn_ < 0.05, MD_hun_amnf_ = 5; MD_hun___amn_ = 7.5), and values of two measurements differed in depersonalization (U_hun___dep_ = 108.132, p_hun_dep_ < 0.05, MD_hun_dep1_ = 3.33; MD_hun_dep2_ = 6.67).

In Table 4 and Table 5, we can see that stress moderately correlated with dissociation in both international (r(483) = 0.436 *p* < 0.001) and Hungarian sample (r(1197) = 0.450, *p* < 0.001). Other variables had low to moderate correlation coefficients with dissociation. Goal-oriented coping (international sample), humor (Hungarian sample) and coping by taking up outer perspective did not have significant correlation coefficients with dissociation.

### 3.2. Moderated Mediation Analysis

**Figure 1:**
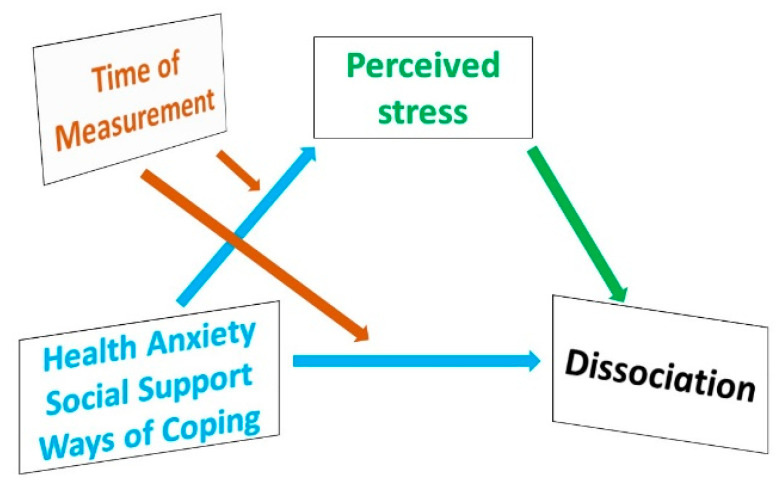
Theoretical model of factors influencing dissociation.

We aimed to examine whether health anxiety, social support and coping (predictor variables) have direct effects on dissociation (dependent variable), or if their effect can rather be explained by mediation through the effect of perceived stress (mediator variable). Time of measurement (in lockdown and after lockdown) may moderate this relationship (moderator variable). Perceived stress as a mediator variable in the model means we wanted to examine the predictor variables’ effect on dissociation through perceived stress. That is to say, we aimed to uncover whether health anxiety, social support and coping affect dissociation by increasing or decreasing perceived stress, or whether they have a direct effect on dissociation in their own right, independent from perceived stress. Our theoretical consideration is demonstrated in Figure 1. Time of measurement as a moderator variable means that time of measurement may moderate the relationship between perceived stress and dissociation and predictor variables and dissociation.

The parameters and model fit measures for the Hayes process analysis are shown in Table 6 and Table 7.

Health anxiety had a positive direct and indirect effect on scale absorption, depersonalization, and the summated score of dissociation in both samples and both measurement times. Its effect on amnesia was positive and only indirect in the Hungarian sample, while in the international sample, it had a positive indirect effect in both measurement times, a positive direct effect in the second measurement time, and a positive tendential direct effect (*p* = 0.051) in the first measurement time.

Considering social support, support by others had no effect on dissociation in the international sample. In the Hungarian sample, there were negative direct effects on depersonalization and amnesia, but only in the second measurement for the latter one. Support by the family showed no effect in the international sample at all, while both direct and indirect negative effects can be seen on each scale regarding the Hungarian sample. There was no direct effect in the case of support by friends, but regarding the international sample it had negative indirect effects on absorption, and in the first measurement on amnesia. While in the Hungarian sample, negative effects can be seen on every scale in the second measurement.

Investigating the effects of coping in the international sample, wishful thinking had positive indirect effects on every scale, and positive direct effects on absorption and, in the first measurement, on depersonalization. Goal-oriented coping had negative indirect effects in the first measurement. Seeking for support had an effect only in the case of absorption and a direct effect in the first measurement. We found a negative indirect effect in the case of think over coping, and tendential positive direct effects of absorption and amnesia (p_abs_ = 0.051, p_amn_ = 0.054) but only in the first measurement. Avoiding seemed to have positive direct effects in every scale of dissociation in the first measurements.

In the Hungarian sample, humor had a negative indirect effect on each scale, and there was a tendential positive effect (*p* = 0.055) in the second measurement of amnesia. Positive thinking was found to have negative indirect effects, while having positive direct effects in the first measurement. There were positive direct and indirect effects on all the scales in the case of distancing. While the outer perspective did not show any significant effect.

The results of direct and indirect effects are summarized in Table 8 and Table 9. The index of moderated mediation was significant only for think over and goal-oriented coping in the international sample.

## 4. Discussion

Our study aimed to investigate how health anxiety, social support, and ways of coping contribute to dissociation directly and through the mediation of perceived stress, moderated by the time of measurement (lockdown). We investigated the effect of perceived stress on different forms (sub-scales) of dissociation.

Depersonalization showed significantly higher values in the second run regarding both Hungarian and international samples; amnesia was higher in the second round in the international sample. That is to say, maladaptive dissociative mechanisms occurred more often with time. Gender differences showed usually reported tendencies for females having higher scores than males [29,56].

Results showing that stress moderately correlated with dissociation in both the international and Hungarian samples are also consistent with the relevant literature [24,25]. Stress was found to relate to dissociation before [31], and also, adaptive forms of dissociation might be a way to cope with stressful situations, because in the context of trauma, it may serve as protection against overwhelming experiences [78]. According to our findings, prolonged stress was related to pathological dissociation (amnesia and depersonalization as well), while absorption as an adaptive coping strategy stayed on a high level over time during the pandemic.

Health anxiety correlated with perceived stress, consistent with previous research [79]. The general positive effect of health anxiety on overall dissociation and all its subscales is not surprising. This result indicates that there is a strong relationship between anxiety and dissociation, directly and through perceived stress as well. This result is consistent with previous research findings about health anxiety and dissociative experiences occurring at the same time [80]. This result emphasizes that reducing anxiety may have a double effect on controlling dissociative experiences: decreased anxiety itself may decrease dissociation directly and through stress reduction.

Results regarding social support were consistent with findings of recent publications reporting that social support moderates anxiety and stress caused by COVID-19 [35,38]. We found several differences between the Hungarian and International samples, as well as regarding measurement time. As to the Hungarian sample, support by the family was so important that it significantly decreased dissociative experiences directly and through decreasing perceived stress as well (considering all scales and both measures). Results reflected that international students were far from their families and in alliance with this we found no significant effects (the direct effect of social support from the family was not significantly decreasing for dissociation, and even slightly increased dissociation through perceived stress). Unlike the support of friends, that seemed to gain more importance for the international students. Again, satisfying social support was proved to relate to less dissociative experiences, consistent with some previous findings [40,41].

As to ways of coping, we found interesting and somewhat contradicting results. Regarding the international sample, the goal-oriented coping strategy had a strong decreasing effect on all dissociation scales in the first measurement, through the mediation of perceived stress. In this sample, wishful thinking proved to increase dissociative experiences overall, and in all its subscales through the mediation of perceived stress, and had a direct increasing effect on absorption and depersonalization. Wu et al [81] found the same adverse effect when examining the effects of COVID-specific wishful thinking of Chinese university students and found that the wishful thinking strategy increased anxiety and also prevented students from adaptive protective behaviors.

As to the Hungarian sample, positive thinking was found to decrease dissociation through decreasing perceived stress, while, just as wishful thinking in the international sample, it had a dissociation-increasing direct effect at the beginning of the pandemic. It may suggest that perceived stress had a strong enough overall effect on dissociation that even if positive thinking (or toxic positivity) may lead to dissociation, shifting from reality, it can still decrease stress and through this effect decreases dissociative experiences. Think over coping showed a similar tendency; it might directly lead to dissociation, but by decreasing stress, it seems to help decrease dissociative experiences. Humor as a strategy seems more confident in decreasing dissociation.

The avoiding coping strategy in the international sample was similar to the distancing coping strategy in the Hungarian sample and showed a similar increasing effect on dissociation directly and indirectly as well.

Our results are consistent with recent research findings [48,52], where problem-focused coping proved to be adaptive in stressful situations, and avoiding or denying things is not a useful strategy for they increase stress, and, in this way, dissociation. Further investigation of the interrelations of the variables is needed, a more complex model could be useful to help understand complicated correlations between the factors examined.

Dissociation can serve as an adaptive coping mechanism in unpredictable stressful situations, maladaptive ways of dissociation may increase the risk of the appearance of dissociative disorders.

Among other negative consequences of increasing dissociative behavior during the COVID-19 pandemic, recent studies report that increased addictive behaviors like alcohol consumption [82] and internet addiction [26] showed correlations with dissociative experiences. We think it is important to find possibilities to decrease the occurrence of dissociative experiences. According to our findings, there are several factors possibly contributing to (such as health anxiety and distancing coping strategy) or alleviating (such as social support) dissociation.

## 5. Conclusions

In respect of limitations, the generalizability of our results is restricted to the population of university students in Debrecen, which was appropriate for testing our model and for investigating the pattern of the interrelation of variables affecting dissociative experiences in the pandemic. On the other hand, other pandemic-related variables were controlled this way (e.g., area-specific factors like the number of cases). We have to take non-response errors into consideration though, that is to say, according to Biddle and Sollis [83], the characteristics (and so answers) of those who do not participate in a particular round of data collection may be different from those who do respond. Although considering the content of the questionnaires, we don’t presume major differences in the psychological contrasts, and the study field and study level of the students represented the whole spectrum of the university, this possibility should still be considered. A further methodological limitation of the study may be that perceived stress is assessed over one month, while health anxiety is assessed over six months.

Several studies have recently reported on the effects of stress on dissociative experiences, mainly in relation to the COVID-19 pandemic [26,27,28,29]. Our results are consistent with these findings, and unlike previous reports, we investigated the effect of perceived stress on the three mechanisms of dissociation with regard to other independent variables with conditional process modeling. Health anxiety, coping and social support appeared to influence dissociation directly and through the mediation of perceived stress. Social support, mainly support from the family and problem-focused coping strategies may decrease the level of stress, this way decreasing dissociative behavior.

When designing interventions and policies for a situation like the COVID-19 pandemic, these specific results should be taken into account.

## Figures and Tables

**Table 1:** Factor loadings for the Hungarian version of WCQ.

	Factor			
	1	2	3	4
item4	0.772			
item3	0.635			
item5	0.499			
item1	0.414			
item15	0.31			
item9		0.565		
item8		0.551		
item16		0.425		
item10		0.399		
item14		0.304		
item7		0.231		
item2			0.984	
item11				0.68
item6				0.50
item13				0.383
item12				0.335

**Table 2:** Descriptive statistics for variables of international sample.

	Minimum	Maximum	Mean_1_ (SD_1_); Mean_2_ (SD_2_)	α_1,_ α_2_
DES	0.00	100.00	19.193(18.052); 23.386(21.120)	0.948; 0.961
DES amnesia	0.00	100.00	11.599(16.172);16.617(20.171)	0.864; 0.909
DES depersonalization	0.00	100.00	13.746(19.880); 17.696(22.441)	0.864; 0.883
DES absorption	0.00	100.00	32.233(24.127); 35.845(27.306)	0.883; 0.919
PSS	0.00	4.00	2.328(.841); 2.179(.912)	0.864; 0.869
SHAI	1.00	3.44	1.963(.443); 1.876(.445)	0.865; 0.860
MSPSS_others	1.00	7.00	4.689(1.951); 4.677(1.906)	0.927; 0.925
MSPSS_family	1.00	7.00	4.988(1.708); 4.812(1.694)	0.909; 0.886
MSPSS_friends	1.00	7.00	4.690(1.732); 4.727(1.171)	0.923; 0.931
WCQ_wishfulthinking	0.00	3.00	2.017(.676); 1.925(0.764)	0.733; 0.783
WCQ_goal-oriented	0.00	3.00	1.844(.719); 1.957(0.753)	0.797; 0.814
WCQ_seeksupport	0.00	3.00	1.285(.783); 1.425(0.823)	0.801; 0.820
WCQ_thinkover	0.00	3.00	1.611(.785); 1.840(0.745)	0.719; 0.711
WCQ_avoid	0.00	3.00	1.544(.750); 1.555(0.753)	0.807; 0.785

Note: Mean_1_(SD_1_, Mean_2_ (SD_2_); α_1_ and α_2_ are for measurement one and two, respectively.

**Table 3:** Descriptive statistics for variables of the Hungarian sample.

	Minimum	Maximum	Mean_1_ (SD_1_); Mean_2_ (SD_2_)	α_1,_ α_2_
DES	0.00	100.00	16.796(15.244); 18.226(15.760)	0.939; 0.938
DES amnesia	0.00	85.00	10.076(13.708); 10.961(13.767)	0.932; 0.822
DES depersonalization	0.00	96.00	10.435(16.621); 12.361(16.808)	0.861; 0.860
DES absorption	0.00	100.00	28.878(15.244); 31.356(22.612)	0.863; 0.870
PSS	0.07	4.00	2.149(.833); 2.050(.863)	0.911; 0.908
SHAI	1.00	4.00	1.912(.420); 1.936(.423)	0.866; 0.862
MSPSS_others	1.00	5.00	4.552(.871); 4.356(1.049)	0.905; 0.915
MSPSS_family	1.00	5.00	3.871(1.138); 3.644(1.259)	0.914; 0.931
MSPSS_friends	1.00	5.00	4.178(1.060); 3.920(1.226)	0.934; 0.935
WCQ_humor	0.00	3.00	1.707(1.103); 1.672(1.130)	
WCQ_positive	0.00	3.00	1.374(.735); 1.402(0.784)	0.697; 0.746
WCQ_distancing	0.00	3.00	0.800(.950); 0.901(613)	0.505; 0.591
WCQ_outer_persepctive	0.00	3.00	1.716(.726); 1.776(0.808)	0.591; 0.673

Note: Mean_1_(SD_1_, Mean_2_ (SD_2_); α_1_ and α_2_ are for measurement one and two, respectively.

**Table 4:** Correlation matrix for the international sample.

	1	2	3	4	5	6	7	8	9	10	11	12	13
1 DES													
2 DES_amnesia	0.800 ^***^												
3 DES_deperson	0.856 ^***^	0.685 ^***^											
4 DES_absorption	0.936 ^***^	0.628 ^***^	0.701 ^***^										
5 Perceived stress	0.436 ^***^	0.268 ^***^	0.351 ^***^	0.473 ^***^									
6. SHAI	0.393 ^***^	0.253 ^***^	0.327 ^***^	0.423 ^***^	0.481 ^***^								
7. MSPSS_others	−0.139 ^**^	−0.59	−0.197 ^***^	−0.107 ^*^	−0.199 ^***^	−0.168 ^***^							
8. MSPSS_family	−0.171 ^***^	−0.116 ^*^	−0.183 ^***^	−0.155 ^***^	−0.287 ^***^	−0.220 ^***^	0.530 ^***^						
9. MSPSS_friends	−0.145 ^***^	−0.048	−0.180 ^***^	−0.119 ^**^	−0.221 ^***^	−0.147 ^***^	0.610 ^***^	0.518 ^***^					
10. WCQ_wishfulthink	0.413 ^***^	0.262 ^***^	0.351 ^***^	0.443 ^***^	0.480 ^***^	0.411 ^***^	−0.025	−0.107 ^*^	−0.006				
11. WCQ_goal-oriented	−0.061	−0.005	−0.069	−0.068	−0.283 ^***^	−0.095 ^*^	0.265 ^***^	0.279 ^***^	0.260 ^***^	0.165 ^***^			
12. WCQ_seeksupport	−0.072	0.049	−0.061	−0.104 ^*^	−0.152 ^***^	−0.048	0.462 ^***^	0.383 ^***^	0.479 ^***^	0.053	0.427 ^***^		
13. WCQ_thinkover	0.106 ^*^	0.111 ^*^	0.099^*^	0.095 ^*^	−0.145 ^***^	0.073	0.169 ^***^	0.205 ^***^	0.145 ^***^	0.283 ^***^	0.540 ^***^	0.398 ^***^	
14. WCQ_avoid	0.163 ^***^	0.123 ^**^	0.160^***^	0.125 ^**^	−0.014	−0.011	−0.163^***^	−0.048	−0.126 ^**^	0.240 ^***^	0.312 ^***^	−0.036	0.293 ^***^

^*^: *p* < 0.05; ^**^: *p* < 0.01; ^***^: *p* < 0.001.

**Table 5:** Correlation matrix for the Hungarian sample.

	1	2	3	4	5	6	7	8	9	10	11	12
1 DES												
2 DES_amnesia	0.809 ^***^											
3 DES_deperson	0.818 ^***^	0.603 ^***^										
4 DES_absorption	0.952 ^***^	0.684 ^***^	0.682 ^***^									
5 Perceived stress	0.450 ^***^	0.340 ^***^	0.372 ^***^	0.438 ^***^								
6. SHAI	0.316 ^***^	0.213 ^***^	0.278 ^***^	0.304 ^***^	0.437 ^***^							
7. MSPSS_others	−0.200 ^***^	−0.185 ^***^	−0.215 ^***^	−0.159 ^***^	−0.265 ^***^	−0.124 ^***^						
8. MSPSS_family	−0.285 ^***^	−0.244 ^***^	−0.245 ^***^	−0.265 ^***^	−0.395 ^***^	−0.222 ^***^	0.526 ^***^					
9. MSPSS_friends	−0.158 ^***^	−0.121 ^***^	−0.186 ^***^	−0.125 ^***^	−0.274 ^***^	−0.208 ^***^	0.562 ^***^	0.481 ^***^				
10.WCQ_humor	−0.039	−0.031	0.008	−0.053	−0.274 ^***^	−0.210 ^***^	0.111 ^***^	0.153 ^***^	0.198 ^***^			
11. WCQ_positive	−0.078 ^**^	−0.066 ^*^	−0.042	−0.070 ^*^	−0.400 ^***^	−0.241 ^***^	0.235 ^***^	0.329 ^***^	0.269 ^***^	0.444 ^***^		
12. WCQ_distancing	0.322 ^***^	0.240 ^***^	0.296 ^***^	0.307 ^***^	0.367 ^***^	0.247 ^***^	−0.095 ^***^	−0.153 ^***^	−0.050	0.032	0.002	
13.WCQ_outer_pers	0.006	−0.005	0.022	0.010	−0.164 ^***^	−0.071 ^*^	0.228 ^***^	0.200 ^***^	0.297 ^***^	0.259 ^***^	0.468 ^***^	0.137 ^***^

^*^: *p* < 0.05; ^**^: *p* < 0.01; ^***^: *p* < 0.001.

**Table 6:** Coefficients of the linear regression of the Hungarian sample.

Dependent Variables	Independent Variables	Indirect Effects		Direct Effects		Index of Moderated Mediation
		Measurement 1	Measurement 2	Measurement 1	Measurement 2	
absorption	SHAI	3.930 ^*^ (2.817; 5.253)	4.617 ^*^ (2.958; 6.441)	5.890 ^*^ (2.772; 9.008)	7.044 ^*^ (1.284; 12.083)	0.687 (−1.012; 2.437)
	MSPSS_others	−0.000 (0.283; −0.565)	4.617 ^*^ (2.958; 6.441)	0.197 (−0.592; 0.989)	−0.597 (−3.161; 1.968)	0.197 (−0.558; 0.971)
	MSPSS_family	−1.149 ^*^ (−1.590; −0.732)	−0.940 ^*^ (−1.5656; −0.316)	−2.322 ^*^ (−3.597; −1.048)	−3.902 ^*^ (−5.944; −1.859)	−0.209 (−0.384; 0.836)
	MSPSS_friends	−0.244 (−0.738; 0.219)	−0.824 ^*^ (−1.486; −0.165)	0.627 (−0.777; 2.032)	−0.289 (−2.452; 1.875)	−0.580 (−1.250; 0.057)
	WCQ_humor	−0.568 ^*^ (−0.980; −0.192)	−0.967 ^*^ (−1.629; −0.352)	0.706 (−1.439; 2.850)	0.104 (−1.108; 1.316)	−0.400 (−1.095; 0.271)
	WCQ_positive	−2.232 ^*^ (−2.994; −1.538)	−2.470 ^*^ (−3.594; −1.434)	4.191 ^*^ (2.157; 6.225)	2.697 (−0.581; 5.976)	−0.238 (−1.243; 0.775)
	WCQ_distancing	3.949 ^*^ (2.954; 5.007)	3.637 ^*^ (2.386; 5.016)	4.887 ^*^ (2.407; 7.367)	5.678 ^*^ (1.725; 9.632)	−0.312 (−1.540; 0.968)
	WCQ_outer_pers	0.142 (−0.450; 0.748)	0.053 (−0.834; 0.939)	0.7034 (−1.270; 2.677)	−1.856 (−5.215; 1.503)	−0.089 (−1.077; 0.903)
depersonalization	SHAI	1.834 ^*^ (1.174; 2.595)	2.155 ^*^ (1.239; 3.254)	4.984 ^*^ (2.499; 7.470)	5.311 ^*^ (0.720; 9.902)	0.321 (−0.497; 1.167)
	MSPSS_others	0.000 (−0.258; 0.271)	0.092 (−0.279; 0.482)	−2.042 ^*^ (−3.449; −0.635)	−2.611 ^*^ (−4.655; −0.567)	0.092 (−0.275; 0.476)
	MSPSS_family	−0.536 ^*^ (−0.788; −0.313)	−0.438 ^*^ (−0.764; −0.149)	−1.414 ^*^ (−2.430; −0.397)	−2.034 ^*^ (−3.663; −0.405)	0.098 (−0.188; 0.402)
	MSPSS_friends	−0.114 (−0.359; 0.110)	−0.385 ^*^ (−0.722; −0.069)	0.068 (−1.052; 1.187)	−0.195 (−1.920; 1.530)	−0.271 (−0.606; 0.415)
	WCQ_humor	−0.265 ^*^ (−0.479; −0.084)	−0.452 ^*^ (−0.782; −0.154)	0.715 (−0.251; 1.681)	1.061 (−0.649; 2.770)	−0.186 (−0.512; 0.139)
	WCQ_positive	−1.042 ^*^ (−1.493; −0.637)	−1.153 (−1.783; 0.607)	2.354 ^*^ (0.732; 3.976)	2.115 (−0.499; 4.729)	−0.111 (−0.608; 0.381)
	WCQ_distancing	1.849 ^*^ (1.216; 2.556)	1.703 ^*^ (1.019; 2.525)	4.091 ^*^ (2.117; 6.065)	7.481 ^*^ (4.334; 10.628)	−0.146 (−0.765; 0.464)
	WCQ_outer_pers	0.066 (−0.221; 0.369)	0.025 (−0.388; 0.452)	0.802 (−1.200; 1.949)	1.366 (−2.928; 2.430)	−0.041 (−0.515; 0.444)
amnesia	SHAI	1.576 ^*^ (0.980; 2.336)	1.851 ^*^ (1.068; 2.839)	1.768 (−0.364; 3.901)	1.136 (−2.803; 5.075)	0.275 (−0.415; 0.986)
	MSPSS_others	0.000 (−0.240; 0.226)	0.079 (−0.250; 0.406)	−0.722 (−1.928; 0.485)	−1.864 ^*^ (−3.617; −0.111)	0.079 (−0.231; 0.410)
	MSPSS_family	−0.460 ^*^ (−0.681; −0.269)	−0.377 ^*^ (−0.674; −0.132)	−1.155 ^*^ (−2.027; −2.083)	−1.993 ^*^ (−3.390; −0.596)	0.0838 (−0.167; 0.339)
	MSPSS_friends	−0.097 (−0.301; 0.096)	−0.328 ^*^ (−0.623; −0.064)	0.484 (−0.477; 1.444)	−0.341 (−1.821; 1.138)	−0.231 (−0.516; 0.028)
	WCQ_humor	−0.229 ^*^ (−0.415; −0.077)	−0.390 ^*^ (−0.694; −0.133)	0.183 (0.665; −0.646)	1.435 (−0.030; 2.901)	−0.161 (−0.4709; 0.106)
	WCQ_positive	−0.893 ^*^ (−1.296; −0.543)	−0.989 ^*^ (−1.572; −0.511)	1.770 ^*^ (0.379; 3.162)	1.211 (−1.032; 3.453)	−0.095 (−0.552; 0.326)
	WCQ_distancing	1.580 ^*^ (1.020; 2.215)	1.456 ^*^ (0.854; 2.179)	3.092 ^*^ (1.396; 4.788)	3.373 ^*^ (0.669; 6.077)	−0.125 (−0.667; 0.398)
	WCQ_outer_pers	0.057 (−0.193; 0.308)	0.021 (−0.334; 0.407)	−0.473 (−1.824; 0.877)	−1.282 (−3.5809; 1.016)	−0.035 (−0.441; 0.372)
Dissociation sum	SHAI	2.447 ^*^ (1.688; 3.293)	2.875 ^*^ (0.582; 4.092)	4.214 ^*^ (1.989; 6.440)	4.4969 ^*^ (0.386; 8.607)	0.427 (−0.651; 1.557)
	MSPSS_others	−0.000 (−0.349; 0.353)	0.122 (−0.373; 0.611)	−0.892 (−2.151; 0.368)	−1.691 (−3,52; 0.139)	0.123 (−0.358; 0.591)
	MSPSS_family	−0.715 ^*^ (−1.004; −0.458)	−0.585 ^*^ (−0.993; −0.195)	−1.630 ^*^ (−2.540; −0.721)	−2.643 ^*^ (−4.101; −1.185)	0.130 (−0.258; 0.536)
	MSPSS_friends	−0.151 (−0.445; 0.144)	−0.512 ^*^ (−0.930; −0.110)	0.392 (−0.609; 1.395)	−0.275 (−1.819; 1.269)	−0.361 (−0.776; 0.030)
	WCQ_humor	−0.354 ^*^ (−0.625; −0.113)	−0.603 ^*^ (−1.035; −0.222)	0.334 (−0.531; 1.199)	1.067 (−0.463; 2.598)	−0.249 (−0.702; 0.168)
	WCQ_positive	−1.389 ^*^ (−1.900; −0.935)	−1.537 ^*^ (−2.268; −0.868)	2.772 (1.320; 4.224)	2.008 (−0.332; 4.348)	−0.148 (−0.798; 0.523)
	WCQ_distancing	2.459 ^*^ (1.787; 3.177)	2.265 ^*^ (1.467; 3.180)	4.023 ^*^ (2.254; 5.793)	5.511 ^*^ (2.690; 8.331)	−0.194 (−1.009; 0.590)
	WCQ_outer_pers	0.088 (−0.287; 0.456)	0.033 (−0.548; 0.609)	0.202 (−1.207; 1.611)	−1.129 (−3.527; 1.269)	−0.055 (−0.672; 0.577)

^*^: effects significantly different from 0, based on 95% confidence interval.

**Table 7:** Coefficients of the linear regression of the Hungarian sample.

Dependent Variables	Independent Variables	Indirect Effects		Direct Effects		Index of Moderated Mediation
		Measurement 1	Measurement 2	Measurement 1	Measurement 2	
absorption	SHAI	3.841 ^*^ (1.963; 5.974)	3.304 ^*^ (1.157; 6.103)	9.230 ^*^ (3.532; 14.928)	19.143 ^*^ (10.651; 27.636)	−0.537 (−2.802; 1.822)
	MSPSS_others	0.079 (−0.213; 0.381)	−0.087 (−0.597; 0.420)	0.043 (−1.376; 1.462)	0.697 (−1.370; 2.765)	−0.166 (−0.730; 0.369)
	MSPSS_family	−0.167 (−0.546; 0.164)	−0.380 (−0.996; 0.159)	0.2938 (−1.210; 1.798)	1.300 (−0.978; 3.578)	−0.213 (−0.836; 0.340)
	MSPSS_friends	−0.420 ^*^ (−0.830; −0.067)	−0.574 ^*^ (−1.254; −0.018)	−0.205 (−1.792; 1.382)	1.379 (−0.994; 3.752)	−0.153 (−0.792; 0.482)
	WCQ_wishfulthinking	3.628 ^*^ (2.010; 5.093)	5.1169 ^*^ (2.720; 7.976)	8.604 ^*^ (4.659; 12.550)	5.835 ^*^ (0.524; 11.147)	1.488 ^*^ (0.121; 3.353)
	WCQ_goal-oriented	−2.561 ^*^ (−4.038; −1.299)	−0.678 (−2.118; 0.515)	−1.765 (−5.691; 2.161)	2.682 (−8.164; 2.377)	1.883 ^*^ (0.524; 3.508)
	WCQ_seeksupport	0.735 (−0.048; 1.598)	0.255 (−0.791; 1.397)	−3.924 ^*^ (−7.353; −0.494)	2.451 (−9.0807; 0.552)	−0.480 (−1.749; 0.771)
	WCQ_thinkover	−1.472 ^*^ (−2.597; −0.622)	−0.939 (−2.273; 0.234)	4.187 (0.694; 7.680)	5.244 (−0.014; 10.502)	0.533 (−0.793; 2.036)
	WCQ_avoid	−0.087 (−0.839; 0.722)	−0.006 (−1.056; 1.219)	3.575 ^*^ (0.269; 6.882)	−4.392 (−1.056; 1.219)	0.081 (−1.224; 1.455)
depersonalization	SHAI	1.393 ^*^ (0.080; 2.913)	1.198 ^*^ (0.046; 2.888)	6.512 ^*^ (1.340; 11.683)	3.923 ^*^ (6.111; 21.527)	−0.195 (−1.186; 0.779)
	MSPSS_others	0.029 (−0.086; 0.161)	−0.031 (−0.248; 0.174)	−0.732 (−2.019; 0.555)	−0.394 (−2.268; 1.480)	−0.060 (−0.309; 0.151)
	MSPSS_family	−0.060 (−0.251; 0.051)	−0.137 (−0.456; 0.053)	0.281 (−1.083; 1.645)	0.771 (−1.295; 2.837)	−0.077 (−0.370; 0.132)
	MSPSS_friends	−0.152 (−0.374; 0.003)	−0.207 (−0.522; 0.029)	−0.918 (−2.357; 0.521)	0.054 (−2.098; 2.206)	−0.055 (−0.311; 0.206)
	WCQ_wishfulthinking	1.410 ^*^ (0.136; 2.710)	1.988 ^*^ (0.189; 3.863)	6.189 ^*^ (2.624; 9.754)	1.139 (−3.660; 5.938)	0.578 (−0.010; 1.511)
	WCQ_goal-oriented	−0.930 ^*^ (−1.939; −0.037)	−0.246 (−1.007; 0.193)	−2.143 (−5.701; 1.415)	−2.963 (−7.740; 1.813)	0.683 (0.004; 1.578)
	WCQ_seeksupport	0.264 (−0.041; 0.725)	0.092 (−0.319; 0.641)	0.225 (−2.883; 3.333)	2.221 (−4.254; 4.477)	−0.173 (−0.763; 0.335)
	WCQ_thinkover	−0.532 ^*^ (−1.219; −0.033)	−0.339 (−0.969; 0.103)	2.121 (−1.045; 5.287)	1.749 (−3.017; 6.515)	0.193 (−0.285; 0.909)
	WCQ_avoid	−0.031 (−0.358; 0.304)	−0.002 (−0.504; 0.474)	4.226 ^*^ (1.211; 7.240)	0.258 (−4.131; 4.648)	0.029 (−0.552; 0.570)
amnesia	SHAI	1.417 ^*^ (0.373; 2.709)	1.219 ^*^ (0.244; 2.571)	4.507 (−0.022; 9.036)	9.654 ^*^ (2.904; 16.404)	−0.198 (−1.172; 0.684)
	MSPSS_others	0.029 (−0.082; 0.154)	−0.032 (−0.237; 0.160)	−0.135 (−1.260; 0.991)	−0.641 (−2.280; 0.998)	−0.061 (−0.300; 0.136)
	MSPSS_family	−0.061 (−0.220; 0.055)	−0.139 (−0.421; 0.056)	0.041 (−1.152; 1.235)	0.053 (−1.754; 1.861)	−0.077 (−0.334; 0.133)
	MSPSS_friends	−0.152 ^*^ (−0.338; −0.014)	−0.207 (−0.487; 0.009)	0.420 (−0.839; 1.679)	−0.474 (−2.357; 1.409)	−0.055 (−0.294; 0.192)
	WCQ_wishfulthinking	1.401 ^*^ (0.454; 2.457)	1.975 ^*^ (0.631; 3.547)	2.809 (−0.314; 5.931)	−0.765 (−4.969; 3.439)	0.575 ^*^ (0.015; 1.431)
	WCQ_goal-oriented	−0.959 ^*^ (−1.744; −0.260)	−0.254 (−0.936; 0.179)	−1.278 (−4.390; 1.834)	−2.443 (−6.621; 1.735)	0.705 ^*^ (0.124; 1.444)
	WCQ_seeksupport	0.270 (−0.014; 0.693)	0.094 (−0.317; 0.564)	1.486 (−1.232; 4.205)	1.407 (−2.412; 5.225)	−0.177 (−0.742; 0.266)
	WCQ_thinkover	−0.552 ^*^ (−1.146; −0.119)	−0.352 (−0.976; 0.078)	2.724 (−0.043; 5.490)	0.485 (−3.679; 4.650)	0.200 (−0.300; 0.836)
	WCQ_avoid	−0.032 (−0.339; 0.271)	−0.002 (−0.452; 0.455)	3.633 ^*^ (0.992; 6.273)	1.129 (−2.716; 4.973)	0.030 (−0.496; 0.571)
Dissociation sum	SHAI	2.217 ^*^ (0.998; 3.695)	1.907 ^*^ (0.583; 3.790)	6.750 ^*^ (2.199; 11.300)	14.205 ^*^ (7.423; 20.987)	−0.310; (−0.634; 1.081)
	MSPSS_others	−0.125 (0.046; 0.233)	−0.050 (−0.357; 0.247)	−0.274 (−1.407; 0.859)	−0.112 (−1.763; 1.538)	−0.096 (−0.439; 0.225)
	MSPSS_family	−0.096 (−0.328; 0.084)	−0.219 (−0.587; 0.087)	0.205 (−0.995; 1.406)	0.708 (−1.111; 2.527)	−0.122 (−0.495; 0.210)
	MSPSS_friends	−0.241 ^*^ (−0.500; −0.035)	−0.330 (−0.730; 0.005)	−0.234 (−1.503; 1.034)	0.320 (−1.577; 2.216)	−0.088 (−0.456; 0.300)
	WCQ_wishfulthinking	2.146 ^*^ (1.025; 3.424)	3.027 ^*^ (1.440; 4.886)	5.867 ^*^ (2.724; 9.011)	2.070 (−2.162; 6.301)	0.880 ^*^ (0.059; 1.962)
	WCQ_goal-oriented	−1.484 ^*^ (−2.449; −0.655)	−0.393 (−1.330; 0.316)	−1.729 (−4.862; 1.405)	−2.767 (−6.973; 1.440)	1.091 ^*^ (0.262; 2.098)
	WCQ_seeksupport	0.423 (−0.016; 1.009)	0.147 (−0.453; 0.869)	−0.737 (−3.475; 3.000)	−0.915 (−4.760; 2.929)	−0.276 (−1.056; 0.484)
	WCQ_thinkover	−0.852 ^*^ (−1.567; −0.307)	−0.543 (−1.352; 0.141)	3.011 ^*^ (0.222; 5.799)	2.493 (−1.704; 6.690)	0.309 (−0.462; 1.238)
	WCQ_avoid	−0.050 (−0.496; 0.427)	−0.003 (−0.646; 0.706)	3.811 ^*^ (1.162; 6.460)	−1.001 (−4.858; 2.855)	0.047 (−0.731; 0.813)

^*^: effects significantly different from 0, based on 95% confidence interval.

**Table 8:** Coefficients of the linear regression of the Hungarian sample.

	Dependent Variables									
	Perceived Stress		Absorption		Depersonalization		Amnesia		Dissociation	
Independent Variable	Coeff	SE	Coeff	SE	Coeff	SE	Coeff	SE	Coeff	SE
Perceived stress			8.611 ^***^	0.842	4.019 ^***^	0.671	3.453 ^***^	0.576	5.361 ^***^	0.601
SHAI	0.456 ^***^	0.53	5.890 ^***^	1.589	4.985 ^***^	1.267	1.768	1.087	4.214 ^***^	1.134
MSPSS_others	−0.093	0.028	0.089	0.833	−2.189 ^**^	0.664	−1.005	0.570	−1.096	0.595
MSPSS_family	−0.127 ^***^	0.020	−2.675 ^***^	0.602	−1.552 ^**^	0.480	−1.343 ^**^	0.412	−1.857 ^***^	0.430
MSPSS_friends	−0.044	0.023	0.416	0.666	0.007	0.531	0.295	0.456	0.239	0.475
WCQ_humor	−0.076 ^***^	0.019	0.228	0.560	0.789	0.446	0.460	0.383	0.492	0.400
WCQ_pos	−0.264 ^***^	0.032	3.880 ^***^	0.964	2.307 ^**^	0.768	1.636 ^*^	0.659	2.608 ^***^	0.688
WCQ_avoid	0.448 ^***^	0.036	5.092 ^***^	1.112	4.982 ^***^	0.886	3.168 ^***^	0.761	4.414 ^***^	0.794
WCQ_out	0.014	0.032	0.140	0.921	0.237	0.734	−0.650	0.630	−0.091	0.657
Constant	2.036 ^***^	0.155	−0.968	4.81	−0.758	3.836	3.285	3.291	0.520	3.434
	R^2^ = 0.411 F(10,1186) = 82.65 ^***^		R^2^ = 0.244 F(11,1185) = 34.836 ^***^		R^2^ = 0.199 F(11,1185) = 26.818 ^***^		R^2^ = 0.130 F(11,1185) = 16.067 ^***^		R^2^ = 0.244 F(11,1185) = 38.685 ^***^	

^*^: effects significantly different from 0, based on 95% confidence interval. ^**^: effects significantly different from 0, based on 99% confidence interval ^***^: effects significantly different from 0, based on 99.9% confidence interval.

**Table 9:** Coefficients of the linear regression of the International sample.

	Dependent Variables									
	Perceived Stress		Absorption		Depersonalization		Amnesia		Dissociation	
Independent Variable	Coeff	SE	Coeff	SE	Coeff	SE	Coeff	SE	Coeff	SE
Perceived stress	-	-	7.310 ^***^	1.483	2.651 ^*^	1.346	2.697 ^*^	1.178	4.219 ^***^	1.184
SHAI	0.525 ^***^	0.087	9.230 ^**^	2.900	6.512 ^*^	2.632	4.507	2.305	6.750 ^**^	2.316
MSPSS_others	0.005	0.020	0.173	0.658	−0.674	0.597	−0.290	0.523	−0.264	0.525
MSPSS_family	−0.031	0.021	0.616	0.689	0.453	0.626	0.075	0.548	0.381	0.551
MSPSS_friends	−0.063 ^**^	0.023	0.147	0.748	−0.706	0.679	0.190	0.595	−0.123	0.598
WCQ_wishful	0.560 ^***^	0.050	7.512 ^***^	1.800	4.465 ^**^	1.634	1.589	1.431	4.522 ^**^	1.438
WCQ_goaloriented	−0.274 ^***^	0.054	−2.248	1.779	−2.496	1.615	−1.708	1.414	−2.151	1.421
WCQ_seeksupport	0.079	0.049	−3.597 ^*^	1.571	0.506	1.426	1.684	1.249	−0.469	1.255
WCQ_avoid	−0.010	0.046	1.398	1.466	3.191 ^*^	1.331	2.176	1.293	2.893 ^*^	1.299
WCQ_thinkover	−0.186	0.050	4.464 ^**^	1.627	2.039	1.477	2.991 ^*^	1.165	2.527 ^*^	1.171
Constant	1.310 ^***^	0.219	−23.218 ^**^	7.325	−14.271 ^*^	6.649	−13.786 ^*^	5.822	−17.091 ^**^	5.850
	R^2^ = 0.456 F(11,471) = 35.910 ^***^		R^2^ = 0.334 F(12,470) = 19.643 ^***^		R^2^ = 0.193 F(12,470) = 9.373 ^***^		R^2^ = 0.137 F(12,470) = 6.217 ^***^		R^2^ = 0.262 F(12,470) = 13.936 ^***^	

^*^: effects significantly different from 0, based on 95% confidence interval. ^**^: effects significantly different from 0, based on 99% confidence interval ^***^: effects significantly different from 0, based on 99.9% confidence interval.

## Data Availability

The data presented in this study are available on request from the corresponding author. The data are not publicly available due to ethical reasons.
